# Inhaled NO Contributes to Lung Repair in Piglets with Acute Respiratory Distress Syndrome via Increasing Circulating Endothelial Progenitor Cells

**DOI:** 10.1371/journal.pone.0033859

**Published:** 2012-03-20

**Authors:** Yuanyuan Qi, Liling Qian, Bo Sun, Lijuan Liu, Panpan Wu, Libo Sun

**Affiliations:** 1 Departments of Pediatrics, Children's Hospital of Fudan University, Shanghai, China; 2 The Key Laboratory of Neonatal Disease, Ministry of Health, Shanghai, China; 3 The Institutes of Biomedical Sciences of Fudan University, Shanghai, China; University of Giessen Lung Center, Germany

## Abstract

**Background:**

Nitric oxide (NO) plays an important role in mobilization of endothelial progenitor cells (EPCs). We hypothesized that inhaled NO (iNO) would induce EPC mobilization and therefore promote lung repair in acute respiratory distress syndrome (ARDS).

**Methodology/Principal Findings:**

Healthy piglets were randomized into four groups (n = 6): Control (Con; mechanical ventilation only); ARDS (established by oleic acid infusion and mechanical ventilation); ARDS plus granulocyte-colony stimulating factor (G-CSF; 10 µg/kg/d subcutaneously); ARDS plus NO inhalation (iNO; 10 ppm). EPCs and mobilizing cytokines were assayed at different time points (baseline, 0, 24, 72 and 168 h) and injury reparation was assessed at 168 h. Compared to the Con group, the levels of EPCs were increased in bone marrow but not in blood in the ARDS group at 24 h. Compared to the ARDS group, inhaled NO induced a rapid elevation in the number of CD34^+^KDR^+^, KDR^+^CD133^+^ and CD34^+^KDR^+^CD133^+^ EPCs in blood (2163±454 vs. 1094±416, 1302±413 vs. 429±244, 1140±494 vs. 453±273 cells/ml, respectively, *P*<0.05), and a reduction in the percentage of KDR^+^CD133^+^ cells in bone marrow. Lung CD34, CD133, VEGF, VEGF receptor 2, endothelial NO synthase mRNA, and VEGF and VEGF receptor 2 protein expression levels were augmented in the iNO group, but not in the G-CSF group, compared to ARDS. Furthermore, iNO treatment reduced vascular permeability, increased pulmonary vessel density, and alleviated pulmonary edema and inflammation compared to ARDS treatment. Plasma VEGF, stromal cell-derived factor-1 (SDF-1) and bone marrow NO_2_^−^/NO_3_^−^ were significantly higher in the iNO group compared to the ARDS group at 72 h.

**Conclusions:**

These results suggest that iNO induces mobilization of EPCs from bone marrow into circulation, contributes to vascular repair, and thereby alleviates lung damage.

## Introduction

Acute lung injury (ALI) and acute respiratory distress syndrome (ARDS) remain important challenges for pediatric intensive care units [1]. Mortality from ARDS approximates 40% despite the advances in supportive and pharmacologic treatment [2]–[4]. Diffuse endothelial damage plays a critical role in the pathogenesis of ALI/ARDS. Data from animal models of ALI demonstrate that up to 50% of lung capillaries are lost and large and small vessels within the lung are damaged during ALI/ARDS [5]. Regeneration of pulmonary endothelium has been suggested as one of the potential therapeutic targets for ARDS [6].

Accumulating evidence suggests that EPCs are mobilized from bone marrow and recruited into tissue to contribute to lung repair after injury. The number of EPCs has been shown to be acutely elevated in circulation, and this increase was associated with the repair of damaged pulmonary endothelium in mice with lung injury [7]. Moreover, patients with low EPC counts reportedly have persistent fibrotic changes in lung tissue after recovery from pneumonia [8], the numbers of EPCs were increased in ALI patients and an adverse outcome was associated with a lower EPC number [9]. Our previous study showed that the level of EPCs was increased in blood and unaltered in bone marrow in animals with moderate lung injury (200 mmHg≤PaO_2_/FiO_2_≤300 mmHg). In contrast, the level of EPCs was unaltered in blood but elevated in bone marrow in animals with severe lung injury (PaO_2_/FiO_2_≤200 mmHg; unpublished observations). These studies suggest that increased circulating EPCs contribute to lung repair in ALI, however the mobilization of EPCs from bone marrow is inhibited in severe lung injury, and thus a lack of sufficient EPCs in circulation might result in an impaired repair process. Therefore, we hypothesize that mobilization of EPCs might provide an effective strategy for restoring pulmonary endothelial function and reducing severity of ARDS.

Several cytokines have been used to mobilize EPCs from bone marrow in cardiovascular diseases [10]. However, currently the use of mobilizing cytokines in ALI is hampered by the fact that most powerful mobilizers, such as granulocyte colony-stimulating factor (G-CSF), also exert a pro-inflammatory effect and may have the potential to promote tumor growth [11]. There is a critical need to search for more safe and effective mobilizers for EPC therapy in ARDS.

Recent studies have demonstrated that endothelial nitric oxide synthase (eNOS) and nitric oxide (NO) play an important role in EPC mobilization [12]–[14]. eNOS transcription enhancer and NO donor supplementation could increase EPC number and improve EPC function in ischemic diseases [15]–[18]. Inhaled NO (iNO) was first used as a vasodilator to selectively dilate pulmonary vessels, and was then shown to have anti-inflammatory effects on the lung [19]. More recent studies have shown that iNO therapy could improve pulmonary angiogenesis. Experimental studies showed that iNO preserved vascular growth and enhanced alveolarisation in newborn rat models of vascular endothelial growth factor receptor (VEGFR2) inhibition, eNOS-deficient and hyperoxia [20]–[22]. Recently, it has been reported that iNO restored vessel density and improved lung structure in neonatal rat model of bronchopulmonary dysplasia (BPD) [23]. Our previous animal studies also indicated that inhaled NO can attenuate the lung injury score in hyperoxic lung injury and ALI animals [24], [25]. However, it is unknown whether iNO can mobilize bone marrow EPCs and consequently promote lung vascular repair.

The aim of this study was to observe the dynamic changes of circulating EPCs, and to further investigate the effect of iNO on EPC mobilization and lung vascular reparation in both acute and recovery phases, using an oleic acid induced ARDS model in young piglets.

## Methods

### Ethics statement

Animal study protocols were approved by the Scientific and Ethics Committees at Children's Hospital of Fudan University (Approval Number: 2007001), and were in compliance with the Chinese national regulations on the use of experimental animals.

### Study animals and protocols

Twenty-four healthy piglets were used in this study. Each animal was anesthetized intramuscularly with 50 mg/kg ketamine and orally intubated with a cuffed endotracheal tube. Mechanical ventilation was initiated in pressure-control ventilation (PCV) mode (Servo 300, Siemens-Elema, Sweden), with a frequency of 15 breaths/min, a positive end-expiratory pressure (PEEP) of 2 cmH_2_O, an inspiration time of 0.33 s, and an inspired oxygen fraction (FiO_2_) of 0.21. The PEEP was adjusted to 6 cmH_2_O after the induction of ARDS, and the ventilator settings were adjusted to provide a tidal volume of 6–8 ml/kg and to maintain target PaO_2_ (>60 mmHg) and PaCO_2_ (30–50 mmHg). The anesthesia was maintained by continuous infusion of ketamine (10 mg/kg/h) and fentanyl (5 µg/kg/h). A central venous catheter was placed into the right external jugular vein for drug administration. A thermodilution catheter (4 Fr, PiCCO plus system, Pulsion Medical Systems) was placed into the right internal carotid artery and connected to a PiCCOplus monitor (Pulsion Medical Systems). The baseline (B) was defined upon stabilization of animals after the operation.

The animals were randomly assigned into each of the following 4 groups (n = 6 each): Con group, where normal animals were ventilated; ARDS group, which was established and treated with mechanical ventilation; G-CSF group, where a subcutaneous injection of G-CSF (a commonly used agent for EPCs mobilizing) was administered to ARDS animals at a dose of 10 µg/kg daily for 7 days; and iNO group, where inhaled NO (10 ppm) was administered to ARDS animals during ventilation and NO gas at 1000 ppm (Noventek) was supplied to the inspiratory line of the ventilator circuit [25]–[27].

ARDS was established by a slow, 30-min intravenous infusion of 0.13–0.15 ml/kg oleic acid (01008; Sigma) suspended in 15 ml of saline via a central venous catheter. ARDS was defined as PaO_2_/FiO_2_≤200 mmHg, with dynamic lung compliance (Cdyn) decreased by more than 50% from its baseline level, and histological evidence of bilateral infiltration of inflammatory cells in the lung (verified by our pilot study in 2 additional piglets) according to previous reports [25], [26]. The moment lung injury was established was defined as treatment time 0 h. There were no differences in the body weight among the four groups, and no difference in PaO_2_/FiO_2_ and Cdyn was found among ARDS, iNO and G-CSF group at time 0 (Table 1, Table S1). Animals were ventilated for 48–72 h and then weaned and fed for recovery until day 7. At different time points (B, 0, 24, 72 and 168 h), blood or bone marrow samples were collected for further analysis.

**Table 1:** General conditions and oxygenation values at 0 h.

Group (n = 6)	Weight (kg)	Sex (M/F)	PaO_2_/FiO_2_ (mmHg)	Cdyn (ml/cmH_2_O/kg)
Con	7.8±0.8	3/3	357±39.0	1.2±0.3
ARDS	8.3±1.4	3/3	133±15.0	0.5±0.2
G-CSF	8.3±0.7	4/2	140±22.1	0.6±0.1
iNO	8.8±0.9	2/4	135±25.3	0.6±0.1

Values are means ± SD. Cdyn, dynamic compliance.

### Lung hemodynamics

Extravascular lung water (EVLW), EVLW index (ELWI) and pulmonary vascular permeability index (PVPI) were determined using the PiCCOplus monitor to evaluate the pulmonary vascular permeability and pulmonary edema at B, 0, 2, 6, 12 and 24 h.

### Flow cytometric analysis

**Figure 1:**
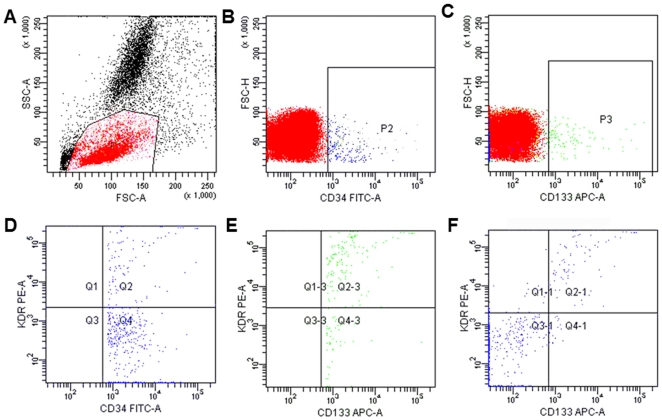
Representative cytograms for the determination of CD34^+^, CD133^+^, CD34^+^KDR^+^, KDR^+^CD133^+^ and CD34^+^KDR^+^CD133^+^ cells from animals. (A) A gate P1 enclosing the mononuclear cells for further selection. P1 selected mononuclear cells are examined for the expression of CD34^+^ cells (B) or CD133^+^ cells(C). (D) CD34^+^ events from P2 are examined further for the expression of VEGFR2 (KDR). (E) CD133^+^ events from P3 are examined for the expression of KDR. (F) CD34^+^ events from P2 are examined for expression of KDR and CD133. The upper right represents events that are CD34^+^KDR^+^CD133^+^.

Blood was collected from the arterial catheter in each animal. Bone marrow was obtained by aspiration of the posterior iliac crest. The EPC levels were assessed by flow cytometry using cell surface antigens in different combinations (CD34^+^KDR^+^, KDR^+^CD133^+^, and CD34^+^KDR^+^CD133^+^ cells) as previously reported [8], [28], [29]. Briefly, before staining with specific monoclonal antibodies, cells were treated with fetal calf serum for 10 minutes, and then the samples were washed with PBS. Then, 100 µl of peripheral blood or bone marrow sample was incubated with 10 µl FITC-conjugated CD34 monoclonal antibodies (mAbs) (348053, BD Biosciences, San Jose, CA, USA), 5 µl of APC-conjugated CD133 mAb (130-090-826, Miltenyi Biotec, Bergisch Gladbach, Germany), and 10 µl of PE-conjugated KDR mAb (FAB357P, R&D Systems, Minneapolis, MN), followed by incubation at 4°C for 30 min. Red cells were lysed and the frequency of EPCs was determined by a two-dimensional forward scatter/fluorescence dot plot analysis of the samples (FACSAria; BD Biosciences, Franklin Lakes, NJ). Isotype controls were used for setting gates and determining the positive/negative boundaries, and the analysis was confirmed by running the FMO (fluorescence-minus-one) controls. After morphological gating to exclude granulocyte and cell debris, we gated CD34^+^ or CD133^+^ cells and then examined the resulting population for dual expression of KDR, and triple positive cells were identified by the dual expression of KDR and CD133 in the CD34 gate (Figure 1A–F). About 500,000 cells were acquired. The levels of EPC were expressed as number of cells per milliliter of blood, and as percentage of total mononuclear cells (MNC) in bone marrow. We did not test the absolute number of EPCs in bone marrow due to the inability to obtain the bone marrow cell counts by the hematology analyzer. The same trained operator, who was blind to the experiment, performed all the tests throughout the study.

### ELISA

The plasma concentrations of VEGF and stromal cell-derived factor-1 (SDF-1) were quantified by commercial ELISA kits. The ELISA was performed according to the manufacturer's instructions.

### Lung processing

At the end of the experiment, the animals were sacrificed with intravenous injection of 10% potassium chloride. After the thorax was opened, lungs and heart were removed en bloc. Lung tissue from the tip of the right mid-lobe was used for wet-to-dry weight (W/D) ratio analysis. The rest of the mid-lobe was immediately frozen in liquid nitrogen for further measurement of RNA and protein. Bronchoalveolar lavage of the right lung was performed with sterile saline via an intracheal tube [30]. The left lung was fixed by perfusion for 30 min via the pulmonary artery with 4% paraformaldehyde in PBS. Lung permeability index (LPI) was measured by determining the ratio of bronchoalveolar lavage fluid protein to plasma protein.

### Morphometric analysis

Sections stained with hematoxylin and eosin were examined by light microscopy for injury patterns and severity as assessed by lung injury scores for edema, neutrophil infiltration, hemorrhage and bronchiole epithelial desquamation [25], [26], [30].

### Immunohistochemistry and vessel density

Fixed lungs were paraffin-embedded and sections were prepared by subsequent deparaffinization and rehydration [27]. The sections were incubated with rabbit polyclonal antibodies against factor VIII (CP039A, 1∶200, Biocare Medical, Walnut Creek, USA), VEGFR2 (KDR) (ab45010, 1∶100; Abcam, Cambridge, UK) and eNOS (ab5589, 1∶100; Abcam, Cambridge, UK) at room temperature for 2 h and then overnight at 4°C. Sections were then incubated with corresponding biotinylated secondary antibodies for 45 min at 37°C followed by avidin-biotin-peroxidase for 45 min at 37°C (Vectastain Elite ABC kit; 1∶200, Vector Laboratories). The sections were then developed with diaminobenzidine (DAB) and H_2_O_2_ and lightly counterstained with hematoxylin. To analyze the vessel density, 5 fields in each slice were randomly selected. The number of vessels was counted using a light microscope at 200× magnification. Results were expressed as number of vessels per field.

### Apoptosis

Lung tissue was analyzed for terminal deoxynucleotidyl transferase dUTP-mediated nick-end labeling (TUNEL) using the DeadEnd Colorimetric TUNEL System (Promega, Madison, WI) according to the manufacturer' protocol. As a negative control, the TdT enzyme was omitted in parallel reactions. All sections were counterstained with hematoxylin after TUNEL staining. The ratio of TUNEL-positive cells to total cells (apoptotic index) was calculated from three random, non-overlapping fields at 400× magnification for each section.

### Quantitative real-time PCR

Total lung RNA was extracted from frozen lung tissue using Trizol Reagent and was reverse-transcribed into cDNA. The primer sequences of target genes VEGF, VEGFR2, eNOS, CD34 and CD133 are described in Table 2. GAPDH was used as a loading control. Real-time PCR was conducted for quantitative analysis using SYBR Green PCR Master Mix as per manufacturer's instructions (TOYOBO, Japan). The endpoint used in the real-time PCR quantification was defined as the PCR cycle number that crossed the signal threshold (Ct). After validation of amplification efficiencies of two genes, quantification of target gene expression was calculated by the comparative Ct method.

**Table 2:** Primers used in quantitative PCR.

Gene	Forward-sense primer	Reverse-antisense primer
VEGFR2	5′CGGAAATGACACTGGAGC3′	5′TTCTGGATACCTCGCACA3′
CD34	5′ACTGCCTGCTGCTGGTCT3′	5′GCTCTGGTGGCTCCTAACAT3′
CD133	5′TCTTTTGTTCCTGCCGTTG3′	5′ACCATAGATGATACCGATGCTTA3′
VEGF	5′CCTTGCTGCTCTACCTCC3′	5′CTCCAGTCCTTCGTCGTT3′
eNOS	5′GTGGTAACCAGCACCTTTGG3′	5′GCAGGAAACACTATTGAAGC3′
GAPDH	5′GGTCGGAGTGAACGGATTTG3′	5′TTCTCAGCCTTGACTGTGCC3′

### Western blot analysis

Lung tissue samples were analyzed for VEGF and VEGFR2 protein expression with western blot analysis. Briefly, forty micrograms of total protein were separated by SDS-PAGE and then transferred to a nitrocellulose membrane by electroblotting. Blots were blocked for 2 h in 10% nonfat dry milk in TBST with 0.1% Tween 20 at room temperature. Immunodetection was performed with rabbit polyclonal VEGF antibody (bs-0279R, 1∶500, Biosynthesis Biotechnology) and rabbit polyclonal VEGFR2 antibody (ab45010, 1∶250, Abcam, Cambridge, UK) at room temperature for 1 h and overnight at 4°C. After the blots were washed to remove unbound antibody, a horseradish peroxidase-conjugated secondary antibody (Jackson Immunoresearch, West Grove, PA) was applied for 1 h at room temperature. After washing, bands were visualized with SuperSignal West Pico chemiluminescent substrate (Thermo Fisher Scientific, Rockford, IL). β-actin (bs-0061R, 1∶500, Biosynthesis Biotechnology) was used as a loading control. Image J software was used to perform densitometry measurements.

### NO assay

NO production in bone marrow plasma was evaluated by measuring the total NO_2_^−^/NO_3_^−^ by Griess reaction (Nanjing Jiancheng Bioengineering Institute).

### Gelatin zymography

Gelatin zymography was performed as described elsewhere [31]. Bone marrow samples were diluted in non-reducing sample buffer and 4 ìl of sample was added to each lane. The samples were electrophoresed in 10% polyacrylamide gels containing 2 mg/ml gelatin. After electrophoresis, gels were washed in 2.5% Triton X-100 for 1 h, incubated for 18 h at 37°C in development buffer, and stained for 3 h with 0.1% Coomassie Brilliant Blue. Gelatinolytic activity of matrix metallopeptidase 9 (MMP-9) was detected as transparent bands on the Coomassie Brilliant Blue-stained gels.

### Statistical analysis

All the continuous data are presented as means and SD. Continuous parametric variables were subjected to ANOVA, followed by post-hoc test of Bonferroni for between-group differences, and by paired samples t test for within-group differences. Nonparametric variables were subjected to Kruskal-Wallis test for differences among groups, followed by Wilcoxon-Mann-Whitney test for between-group differences. A *P* value<0.05 was regarded as statistically significant.

## Results

### Inhaled NO induces rapid mobilization of bone marrow EPCs into circulation

In the G-CSF group, the WBC counts gradually increased compared to the ARDS group (Table 3). Moreover, the total WBC counts fell dramatically at 24 h in ARDS group, G-CSF and iNO increased the WBC counts compared with ARDS (Table 3). There was no difference in the level of EPCs (CD34^+^KDR^+^, KDR^+^CD133^+^, and CD34^+^KDR^+^CD133^+^ cells) among the four groups either in peripheral blood or in bone marrow at baseline (Table 4). In the blood, the numbers of various EPC types in the ARDS group did not differ from those in the Con group at any time point (Table 4). These results suggest impairment in EPC mobilization from bone marrow into circulation in ARDS. Inhaled NO significantly increased circulating EPC numbers (in all three EPC typs) compared to ARDS at 24 h, and this increase was sustained up to 168 h for CD34^+^KDR^+^ and KDR^+^CD133^+^ EPCs. The percentage of CD34^+^KDR^+^/MNC was also higher in iNO than ARDS at 24 and 72 h (Table S2). No differences were detected in the EPC numbers between G-CSF and ARDS at 24 h. However, we observed an elevation in the number of CD34^+^KDR^+^ cells at 72 h and in the number of all types of EPCs at 168 h in G-CSF compared to ARDS, along with elevation in the percentage of CD34^+^KDR^+^ at 72 h and CD34^+^KDR^+^ and KDR^+^CD133^+^ at day 7 (Table S2). The increase of CD34^+^KDR^+^ cells in the G-CSF group compared to ARDS group was more pronounced than that of the iNO group compared to ARDS group at 168 h.

**Table 3:** Total numbers of WBCs (×10^9^/L) in different groups at different time points.

Group	Baseline (n = 6)	0 h (n = 6)	24 h (n = 6)	72 h (n = 5–6)	168 h (n = 5–6)
Con	19.1±3.5	18.4±3.3	19.2±4.1*	18.3±3.3	17.1±1.7*
ARDS	20.9±6.4	17.6±3.1	12.2±1.8†	15.9±1.5†	13.7±1.4†
G-CSF	18.1±3.6	16.8±2.8	30.5±9.1** †	38.3±21.5** †	42.6±29.8** †
iNO	21.3±5.9	18.6±4.4	19.5±8.1*	19.9±5.6	16.8±3.6

Values are means ± SD.

* *P*<0.05 vs. ARDS;

** *P*<0.01 vs. ARDS;

† *P*<0.05 vs. Baseline.

**Table 4:** EPC number in peripheral blood from animals of different groups.

	Group	Baseline (n = 6)	0 h (n = 6)	24 h (n = 6)	72 h (n = 5–6)	168 h (n = 5–6)
CD34^+^KDR^+^ cells(cells/ml)	Con	1164±799	955±536	1238±643	1166±685	1032±634
	ARDS	1145±703	975±637	1094±416	1176±603	1024±330
	G-CSF	1160±440	966±539	1430±1011	4097±1468*	5715±3505*
	iNO	1151±277	955±375	2163±454*	2198±671*	2100±794* †
CD34^+^KDR^+^CD133^+^ cells (cells/ml)	Con	424±314	478±381	497±268	400±216	455±287
	ARDS	404±275	423±278	453±273	366±191	313±198
	G-CSF	416±225	377±127	451±230	797±323	901±506*
	iNO	435±125	382±74	1140±494* †	822±321	739±303
KDR^+^CD133^+^ cells(cells/ml)	Con	486±364	509±375	544±303	498±276	507±227
	ARDS	459±300	356±202	429±244	468±249	406±281
	G-CSF	446±264	360±182	553±375	867±488	1420±572*
	iNO	514±175	442±113	1302±413* †	1056±347*	952±272*

Values are means ± SD.

* *P*<0.05 vs. ARDS;

† *P*<0.05 vs. G-CSF.

In bone marrow, the percentages of all three types of EPCs were higher in ARDS versus Con at 24 h. Interestingly, iNO and G-CSF reduced the percentage of KDR^+^CD133^+^ cells in bone marrow at 24 h compared to the ARDS. The percentage of CD34^+^KDR^+^CD133^+^ EPCs was also lower in the G-CSF group relative to ARDS group. There was a trend towards a reduction in the percentage of bone marrow CD34^+^KDR^+^ cells in both iNO and G-CSF groups compared to ARDS at 24 h (Table 5).

**Table 5:** EPC level in bone marrow from animals of different groups.

	Group	Baseline (n = 6)	24 h (n = 6)	72 h (n = 5–6)
CD34^+^KDR^+^ cells/MNC, %	Con	0.014±0.005	0.017±0.005	0.014±0.004
	ARDS	0.014±0.009	0.050±0.020†	0.018±0.005
	G-CSF	0.013±0.009	0.021±0.014	0.017±0.013
	iNO	0.012±0.006	0.026±0.014	0.015±0.008
CD34^+^KDR^+^CD133^+^ cells/MNC, %	Con	0.008±0.003	0.011±0.003	0.007±0.003
	ARDS	0.007±0.004	0.022±0.007†	0.016±0.006
	G-CSF	0.008±0.005	0.009±0.004*	0.004±0.003
	iNO	0.008±0.004	0.012±0.005	0.007±0.004
KDR^+^CD133^+^ cells/MNC, %	Con	0.008±0.003	0.011±0.005	0.011±0.006
	ARDS	0.007±0.004	0.028±0.012†	0.014±0.005
	G-CSF	0.009±0.004	0.011±0.006*	0.005±0.003
	iNO	0.009±0.004	0.013±0.007*	0.008±0.003

Values are means ± SD. MNC, mononuclear cells.

* *P*<0.05 vs. ARDS;

† *P*<0.05 vs. Con.

### Effects of iNO on plasma and bone marrow EPC mobilizing cytokines during lung injury

There was no significant difference in plasma SDF-1 and VEGF concentrations between groups at baseline (Table 6). The plasma VEGF and SDF-1 levels were not change significantly different between ARDS and Con at any time point. In comparison with the ARDS group, the iNO group showed an elevation in the plasma VEGF (256±81.1 vs. 478±63.3 ng/l, p<0.05, respectively) and SDF-1 (4.3±1.3 vs. 6.0±1.2 µg/l, p<0.05, respectively) at 72 h. G-CSF treatment increased the VEGF concentration but did not affect the level of SDF-1 at any time point.

**Table 6:** Cytokine concentrations in plasma evaluated by ELISA.

Group	VEGF (ng/l)					SDF-1 (ìg/l)				
	Baseline (n = 6)	0 h (n = 6)	24 h (n = 6)	72 h (n = 5–6)	168 h (n = 5–6)	Baseline (n = 6)	0 h (n = 6)	24 h (n = 6)	72 h (n = 5–6)	168 h (n = 5–6)
Con	264±47.3	288±60.9	316±98.5	292±27.7	248±110	4.7±0.9	4.4±0.9	3.9±0.8	4.4±1.0	4.2±1.2
ARDS	299±33.7	294±54.8	314±116	256±81.1	257±83.9	4.4±0.5	5.4±0.8	4.1±1.2	4.3±1.3	4.0±1.2
G-CSF	279±29.0	316±33.3	309±63.3	387±45.1‡	293±76.5	5.1±1.3	3.9±1.3	4.1±1.2	4.3±0.8	4.4±1.4
iNO	308±27.8	331±40.1	357±44.9	478±63.3* § ‡	352±31.9	5.1±1.2	5.0±1.2	5.1±0.9	6.0±1.2* †	5.8±0.6

Values are means ± SD.

* *P*<0.05 vs. ARDS;

§ *P*<0.05 vs. Con;

† *P*<0.05 vs. Baseline,

‡ *P*<0.01 vs. Baseline.

**Figure 2:**
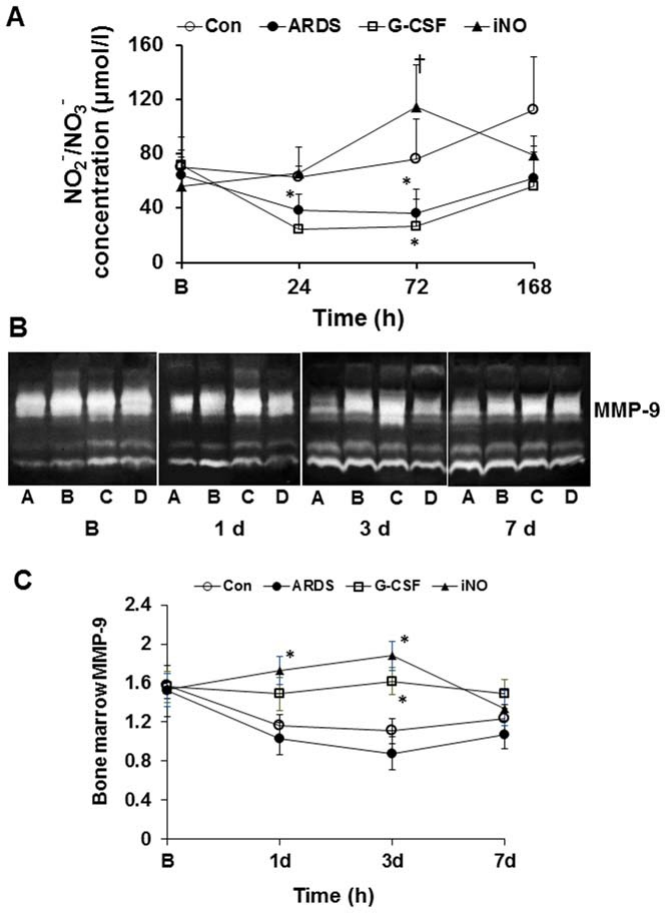
Effects of iNO on NO_2_^−^/NO_3_^−^ and MMP-9 in bone marrow. (A) In the iNO group, the NO_2_^−^/NO_3_^−^ concentration was increased at 72 h compared to the ARDS group. In the ARDS group, the NO_2_^−^/NO_3_^−^ level decreased at 24 and 72 h compared to baseline. (B) MMP-9 expression measured by Zymography in bone marrow, A–D represent ARDS, G-CSF, iNO and Con, respectively. (C) Inhaled NO enhanced the MMP-9 expression at day 1 and 3. (* *P*<0.05 vs. B; † *P*<0.05 vs. ARDS group. n = 5–6 in each group).

No significant difference was observed in bone marrow plasma NO_2_^−^/NO_3_^−^ concentrations between groups at baseline. ARDS animals demonstrated a marked decline in the concentrations of NO_2_^−^/NO_3_^−^ at 24 and 72 h (Figure 2A). The NO_2_^−^/NO_3_^−^ level in bone marrow was higher in iNO animals versus ARDS at 72 h (Figure 2A). However, NO_2_^−^/NO_3_^−^ was not increased in bone marrow in the G-CSF group.

Gelatin zymography analysis demonstrated that, compared to ARDS, iNO augmented the expression of MMP-9 in bone marrow at 24 and 72 h, while bone marrow MMP-9 expression was increased in the G-CSF group at 72 h (Figure 2B and C).

### Inhaled NO restores lung vascular growth and improves lung histology via EPC mobilization

There was a trend towards improvement in ELWI and PVPI in the iNO and G-CSF groups, whereas the ARDS group showed deterioration, during the first 12 h. The values of ELWI and PVPI were dramatically decreased in iNO compared to ARDS (13.2±1.6 vs. 24.7±9.2 and 4.6±0.6 vs. 6.1±1.1, respectively) at 24 h. The LPI was lower in iNO than in ARDS. The Lung W/D ratio was also decreased in the iNO group compared to the ARDS group at day 7 (Table 7).

**Table 7:** Lung injury score, LPI and W/D ratio in different groups.

Group	Edema	Neutrophil infiltration	Hemorrhage	Bronchiole epithelial desquamation	W/D	LPI
Con (n = 6)	0.2±0.4	0.3±0.5	0.2±0.4	0.4±0.5	4.8±0.4	2.5±0.9
ARDS (n = 5)	1.3±0.9	2.0±1.2	1.5±1.2	1.4±0.6	5.9±0.3	11.4±5.0
G-CSF (n = 5)	0.4±0.5	2.3±0.9	0.3±0.5	0.6±0.4*	5.3±0.2	4.3±2.4*
iNO (n = 6)	0.3±0.5*	1.0±0* †	0.2±0.4	0.5±0.5*	5.2±0.3*	3.7±1.5*

Values are means ± SD. W/D, wet-to-dry lung weight ratio; LPI, lung permeability index.

* *P*<0.05 vs. ARDS,

† *P*<0.05 vs. G-CSF.

**Figure 3:**
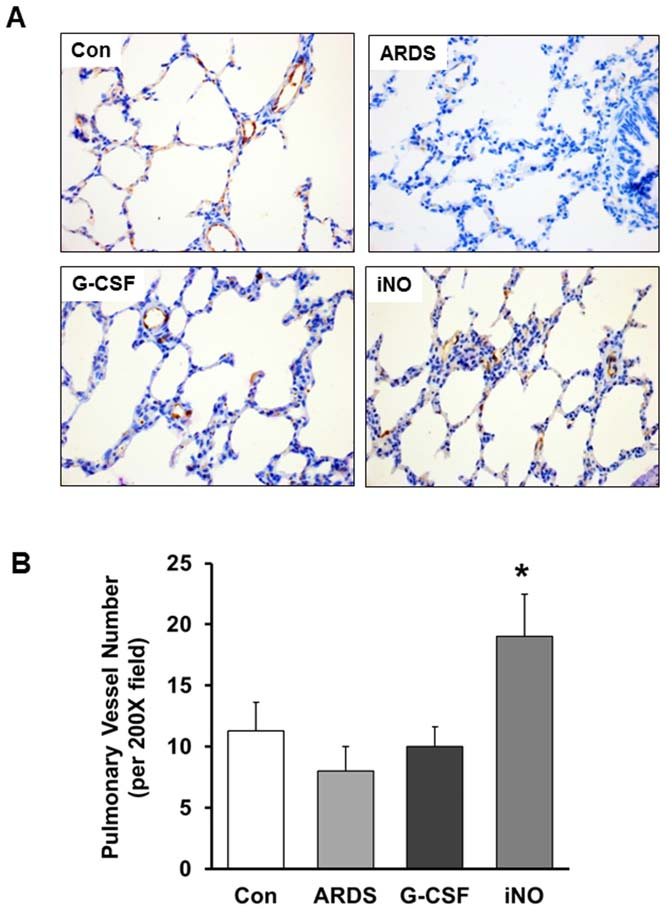
Vessel density of animals in different groups at day 7. (A) Representative photomicrographs (×400) illustrating Factor VIII staining to identify blood vessels in the lung. (B) The number of vessels per high-powered field is significantly higher in the iNO group relative to the ARDS group. (* *P*<0.05 vs. ARDS group, n = 5–6 in each group).

The iNO animals exhibited higher factor VIII-positive vessel density than the ARDS group (Figure 3A and B). G-CSF treatment did not significantly change the vessel density compared to the ARDS group.

**Figure 4:**
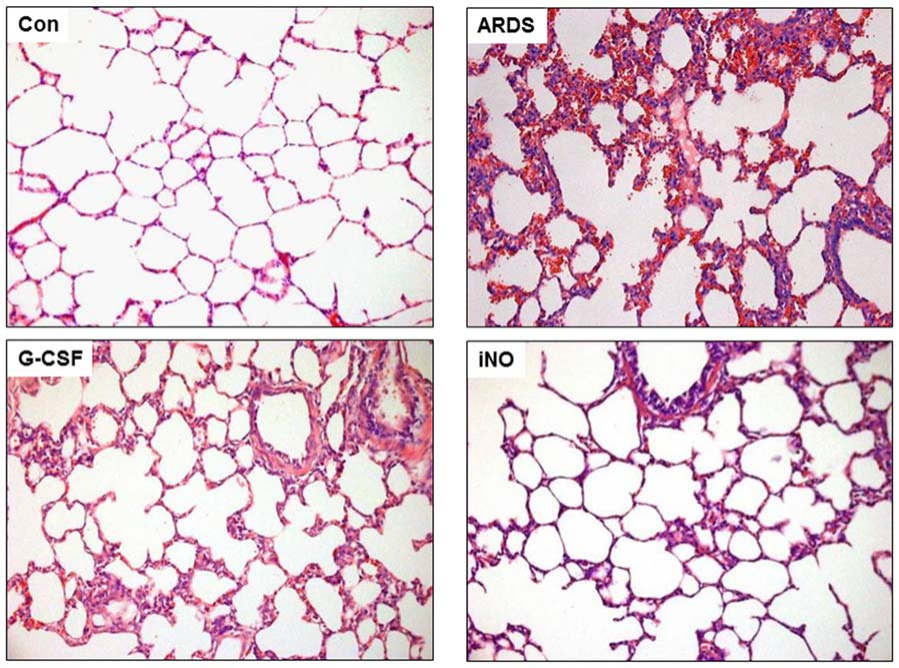
Lung histology from animals of different groups at day 7. Lung sections were stained with hematoxylin and eosin (HE) and inflammation cell infiltration, edema and hemorrhage were visualized at 200× magnification. Oleic acid and mechanical ventilation resulted in atelectatic, congested and edematous lung, with hemorrhagic spots in the lung. There was significantly alleviated edema, neutrophil infiltration and alveolar epithelial desquamation in the iNO group compared to the ARDS group.

Lung histology from the ARDS group was characterized by atelectatic, congested and edematous with hemorrhagic spots (Figure 4). As indicated by the lung injury score, there was mildly less severe hemorrhage, but dramatically alleviated edema, neutrophil infiltration and alveolar epithelial desquamation in the iNO group compared to the ARDS group (Table 7). There was also attenuated alveolar epithelial desquamation in the G-CSF group. However, lung inflammation was more prominent in the G-CSF than the iNO group.

**Figure 5:**
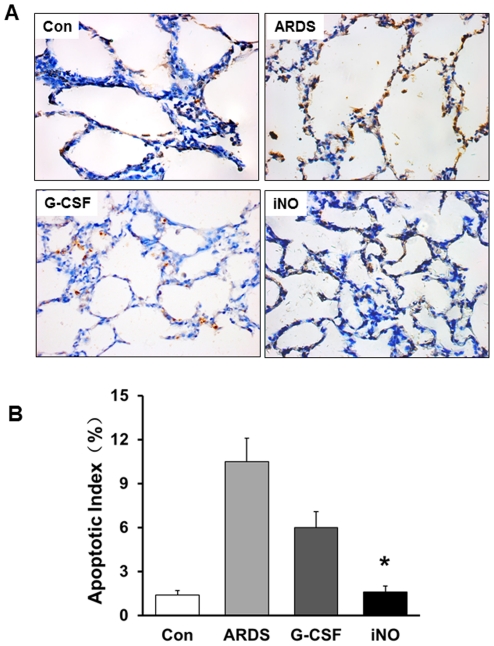
Effects of iNO on apoptosis of lung tissue cells at day 7. (A) Representative images of lung sections showing the TUNEL staining. (B) Bar graph showed the ratio of TUNEL-positive cells was decreased in the iNO group compared to the ARDS group. (* P<0.05 vs. ARDS group, n = 5–6 in each group).

TUNEL staining exhibited little or no labeling in Con animals, while more cells with intense labeling were present in lungs of ARDS group animals. The apoptotic index was markedly reduced in the iNO group compared to the ARDS group (Figure 5).

**Figure 6:**
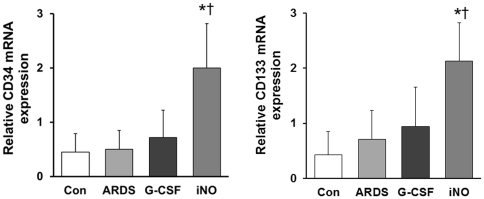
Lung CD34 and CD133 mRNA expression at day 7. In the iNO group, lung CD34 (A) and CD133 (B) mRNA levels were increased compared to the Con and ARDS groups. (* *P*<0.05 vs. ARDS group; † *P*<0.05 vs. Con group; n = 5–6 in each group).

CD34 mRNA level did not change greatly in the ARDS group, as compared to Con. CD34 mRNA expression was 4-fold greater in the iNO group than in the ARDS group. CD133 mRNA expression was also elevated in the iNO group compared to the ARDS group (Figure 6). No difference was found in CD34 and CD133 mRNA expression between G-CSF and ARDS groups.

### Inhaled NO increases VEGF, VEGFR2, and eNOS expression

**Figure 7:**
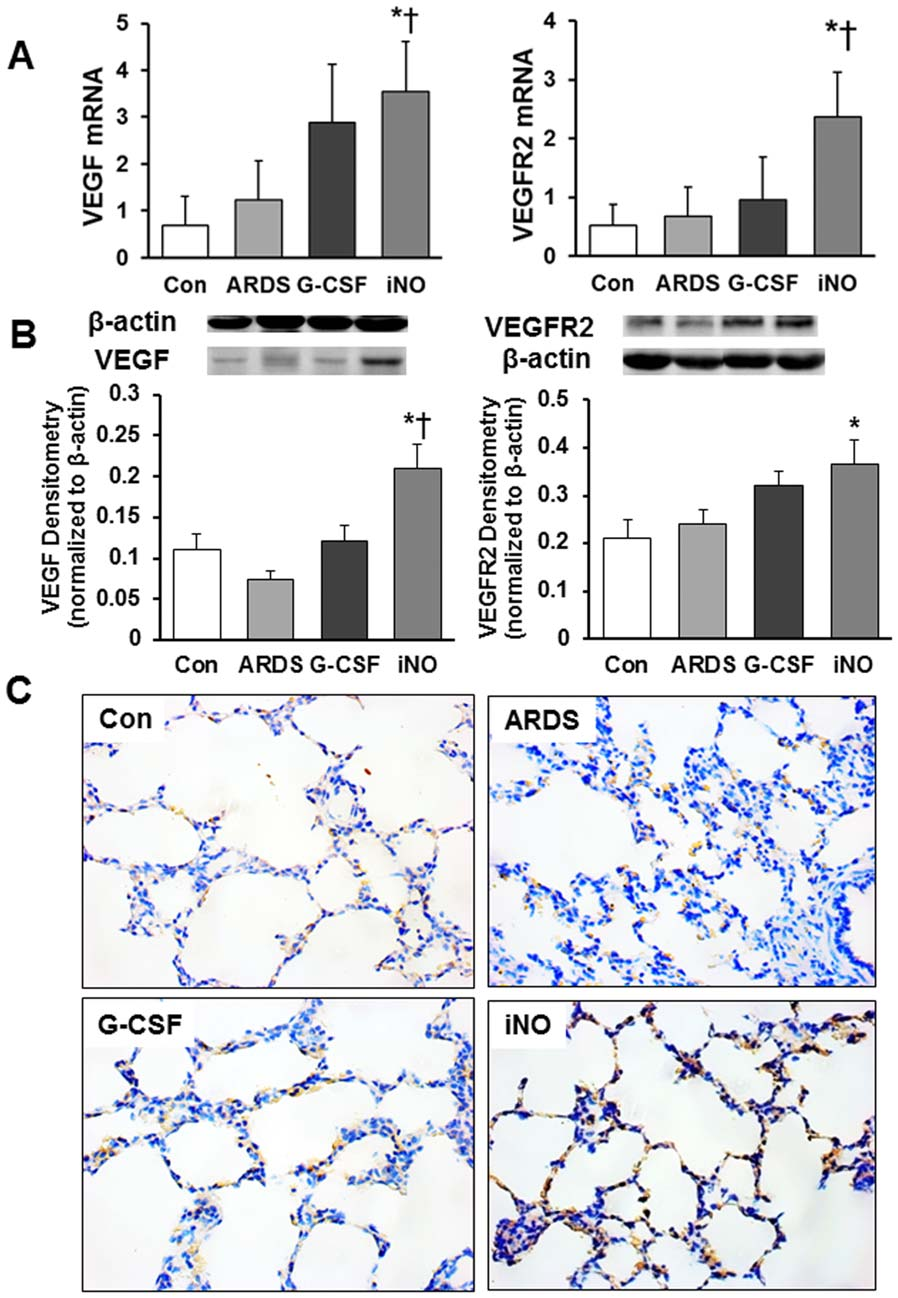
Effects of iNO on lung VEGF and VEGFR2 expression at day 7. (A) VEGF and VEGFR2 mRNA expression levels (normalized to GAPDH) were increased in the iNO group compared to the ARDS and Con groups. (B) Representative blots for VEGF and VEGFR2 (top) and bar graph for band densitometry (bottom). (C) Representative immunohistochemical images showed enhanced VEGFR2 expression in the iNO group compared to the ARDS group. (* *P*<0.05 vs. ARDS group; † *P*<0.05 vs. Con group. n = 5–6 in each group).

**Figure 8:**
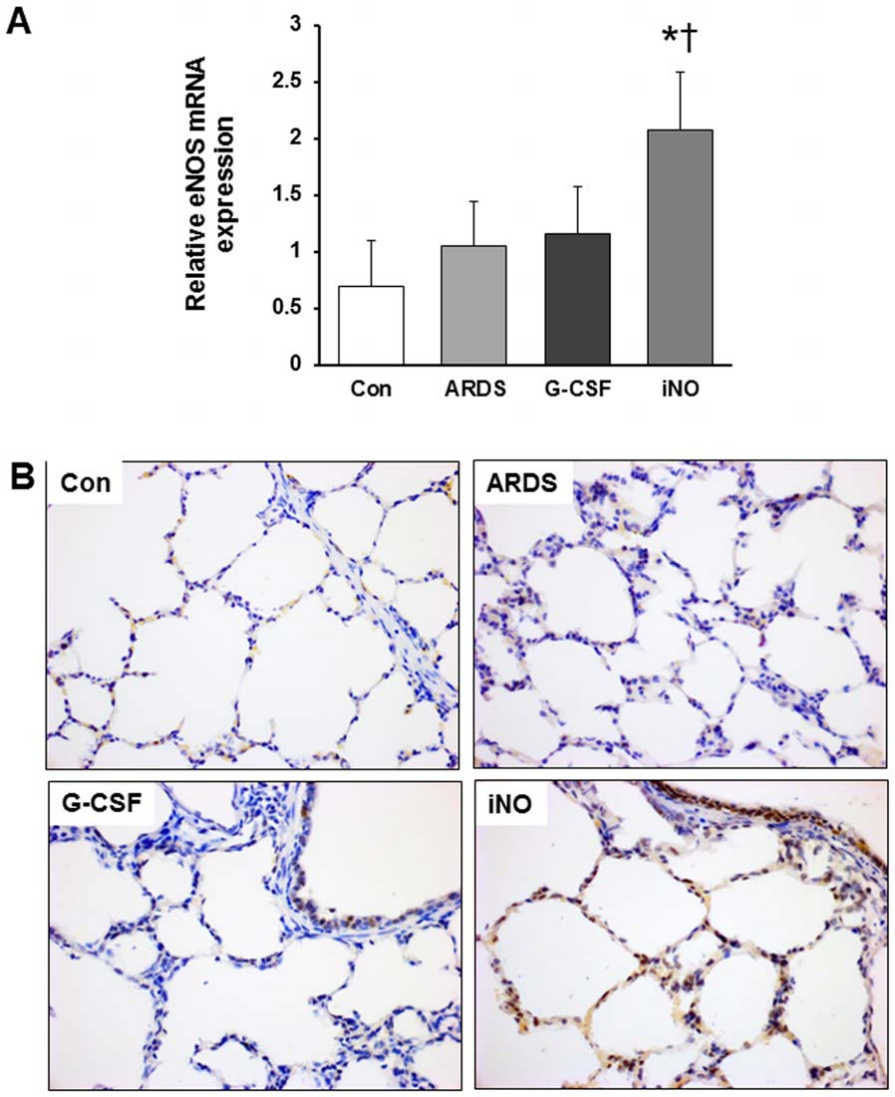
Effects of iNO on lung eNOS expression at day 7. (A) eNOS mRNA expression (normalized to GAPDH) increased in the iNO group compared to the Con group. (B) Representative immunohistochemical images show enhanced eNOS expression in iNO relative to ARDS. (* P<0.05 vs. Con group. n = 5–6 in each group).

There was no difference in VEGF and VEGFR2 mRNA expression between ARDS and Con groups. Lung VEGF and VEGFR2 mRNA expression was significantly higher in iNO animals, but not G-CSF animals, compared to ARDS (Figure 7A). As determined by western blot analysis, lung VEGF and VEGFR2 protein was elevated in iNO relative to ARDS (Figure 7B). Moreover, immunochemistry of lung sections also showed stronger VEGFR2 staining in the iNO group than in the ARDS group (Figure 7C). Increased eNOS mRNA expression was also noted in the iNO group, as reflected by the immunohistochemical staining (Figure 8A and B).

## Discussion

Diffuse endothelial damage is a hallmark of ARDS. Potential therapies targeted at repair of endothelial damage have not been thoroughly explored. It is believed that EPCs could be a prognostic biological marker and that EPC mobilization could ameliorate lung injury. However, little is known about the dynamics of EPC mobilization and homing during the course of ARDS or the long term therapeutic efficacy of EPC mobilization on lung vascular and tissue repair.

To date, no consensus has yet been reached on the definition of EPCs or methods for their identification. The CD34^+^KDR^+^CD133^+^ cells are widely accepted as circulating EPC. Flow cytometric analyses of three surface antigens and corresponding subpopulations of EPCs (CD34^+^KDR^+^, KDR^+^CD133^+^ and CD34^+^KDR^+^CD133^+^ cells) have been used for EPCs enumeration, and have been shown to correlate with severity of disease state in many studies [8], [28], [32]. Subsequently, some studies indicated different phenotype of EPC, for example, Case et al [33] reported that CD34^+^CD133^+^VEGFR2^+^ cells do not spontaneously form capillary-like structures with lumens. Further studies are required regarding the specific phenotypic of EPC to develop the consensus definition on EPC and to understand its role in vascular repair. Despite the controversy, the triple-positive cell population was still used for the enumeration of EPC in recent published articles [34], [35]. Therefore, we still chose the combinations of antigens to detect different EPC populations.

Previous studies have shown that bone marrow EPCs are released into peripheral blood during ALI/ARDS [7], [8], and notably, an insufficient number of circulating EPCs has been correlated with adverse outcomes in ARDS [8], [9]. Recent evidence suggests that intravenous EPC transplantation promotes the integrity of lung vessels after lung injury [36]–[38]. However, the clinical application of cell-therapy is limited by several factors, such as the scarcity of circulating EPCs and the technical difficulty of purifying EPCs. Exogenous mobilization of EPCs from bone marrow may provide a less cumbersome and potentially more effective strategy to achieve therapeutic angiogenesis. G-CSF is one of the prototypical mobilizing cytokines that has been used for stem cell and EPC mobilization in cardiovascular diseases. NO-mediated EPC release into circulation and subsequent angiogenesis has been described previously. Inhaled NO has been demonstrated to improve oxygenation and preserve vascular growth of lung in a BPD model [21]–[23]. In the present study, we used iNO treatment as a strategy to stimulate the mobilization of EPCs and compared its effects with G-CSF. Our results showed that G-CSF mobilized EPCs from the bone marrow after 3 days' administration, which was consistent with previous reports [39], but iNO treatment increased the number of EPCs more rapidly than G-CSF. Higher levels of EPCs in the blood were observed on day 7 in the G-CSF group, possibly due to the 7 day administration of G-CSF, while NO inhalation was only administered for 48–72 h along with mechanical ventilation. These findings suggest that iNO has a profound effect on EPC mobilization.

It is well established that endothelial-active cytokines, such as VEGF and SDF-1 induce EPC mobilization [10]. Previous studies have demonstrated that activation of VEGF/eNOS signaling induces bone marrow NO production and subsequent up-regulation of MMP-9 expression [40]. Of interest here, plasma concentrations of SDF-1 and VEGF were elevated in the iNO group. Moreover, we also found that iNO increased MMP-9 expression in bone marrow. These data suggest a possible mechanism whereby iNO acts to increase the mobilization factors, SDF-1 and VEGF, and therefore promote EPC mobilization. However, the precise mechanism by which inhaled NO enhances EPC mobilization and improves the lung repair remains to be defined.

Mobilization of EPCs might result in accelerated regeneration and a promotion in the repair process of injured vessels [41]. We found that iNO promoted the integrity of pulmonary endothelium, increased the vascular density and alleviated the lung histological injury compared to ARDS. Multiple lines of evidence suggest that EPCs are recruited to sites of injury and participate in the tissue repair [42]. Here, we showed that iNO treatment augmented the mRNA expression of EPC surface markers CD34 and CD133 in lung tissue. VEGF is an important growth factor in vasculogenesis and angiogenesis during lung development. Moreover, accumulating evidence suggests that VEGF and eNOS play critical roles in EPC mobilization, adhesion and incorporation into damaged vessels [12], [43], [44]. VEGF exerts its biological effect on vascular endothelium through specific receptors, especially VEGFR2. In accordance with previous studies [20], [23], [45], we found that iNO up-regulated VEGF and VEGFR2 expression in the lung on day 7, compared to the ARDS group. Our data also showed that iNO enhanced the lung tissue eNOS expression. Collectively, our data suggests that inhaled NO promotes angiogenesis and reendothelization of pulmonary vascular endothelium, thereby improving lung repair. The beneficial effects of iNO are likely due to the promotion of mobilization and homing of EPCs.

One of the major limitations of this study is that we did not measure the EPC number in lung tissue and therefore were unable to evaluate the incorporation of circulating EPCs into the lung. However, we determined the CD34 and CD133 mRNA expression levels, which partially reflects the EPC levels in lung. The effects of iNO on the VEGF/eNOS or SDF/CXCR4 signal pathways of EPCs homing and recruitment have not been demonstrated. Some studies showed that multiple pathways affect trafficking of EPCs to sites of injury. More studies are needed to determine the role of iNO on homing and recruitment of EPCs to lung tissue. In addition, studies have shown that the role of EPCs is not only to repair or regenerate damaged vasculature, but also to produce several cytokines, such as IL-10. Cytokine levels were not examined in this study.

In conclusion, we have provided substantial evidence demonstrating that inhaled NO mobilizes bone marrow EPCs into circulation and attenuates damage of lung alveolar-capillary barrier in ARDS. These findings suggest a feasible therapy for ARDS. The mechanism of EPC mobilization, homing to injured lungs and subsequent involvement in lung repair is a more complicated process, requiring further investigation.

## Supporting Information

Table S1 Blood gas values and PaO_2_/FiO_2_ during ventilation time.
(DOC)

Table S2 The number of EPCs/MNC in peripheral blood at different time points.
(DOC)
