# Supplementary material for: Inhaled NO Contributes to Lung Repair in Piglets with Acute Respiratory Distress Syndrome via Increasing Circulating Endothelial Progenitor Cells
Source: PLoS One. 2012 Mar 20;7(3):e33859. doi: 10.1371/journal.pone.0033859 (PMC3309020; doi:10.1371/journal.pone.0033859)
Supplement: Table S1 — Blood gas values and PaO2/FiO2 during ventilation time. (DOC) [file pone.0033859.s001.doc]

Table S1: Blood gas values and PaO_2_/FiO_2_ during ventilation time

|  | Group (n=6) | 0 h | 2 h | 6 h | 12 h | 24 h |
| --- | --- | --- | --- | --- | --- | --- |
| PaCO_2_ (mmHg) | Con | 33.6±1.7 | 31.4±3.5 | 31.4±3.1 | 36.0±5.4 | 36.5±6.7 |
|  | ARDS | 54.0±9.3 | 46.3±10.6 | 44.0±16.7 | 35.8±9.5 | 39.8±8.6 |
|  | G-CSF | 48.0±6.6 | 42.6±7.7 | 35.0±7.3 | 34.8±5.8 | 33.8±1.0 |
|  | iNO | 51.2±2.3 | 43.8±3.3 | 35.8±2.4 | 34.2±4.9 | 36.4±4.8 |
| PaO_2_ (mmHg) | Con | 75.3±7.4 | 72.5±7.9 | 71.7±7.2 | 66.3±5.1 | 63.2±3.1 |
|  | ARDS | 46.8±9.0 | 85.6±30.5 | 72.4±30.8 | 86.5±27.1 | 89.8±32.1 |
|  | G-CSF | 44.2±6.8 | 73.0±26.9 | 72.6±18.1 | 95.4±19.2 | 60.2±9.4 |
|  | iNO | 43.4±11.2 | 79.0±12.2 | 73.0±7.5 | 89.2±17.9 | 68.0±5.1 |
| BE (mmol/L) | Con | 2.4±2.0 | 1.8±1.6 | 1.8±2.1 | 0.8±1.0 | 0.6±0.8 |
|  | ARDS | -6.2±5.0 | -3.4±2.5 | 0.6±1.2 | 2.0±1.7 | 2.3±1.6 |
|  | G-CSF | -4.4±3.8 | -2.2±1.8 | 1.2±1.0 | 2.3±1.5 | 2.5±2.1 |
|  | iNO | -4.8±4.1 | -2.4±2.0 | 1.4±0.8 | 2.6±2.0 | 3.4±2.8 |
| PaO_2_/FiO_2_ (mmHg) | Con | 357±39.0 | 345±30.8 | 337±23.6 | 312±31.3 | 301±14.2 |
|  | ARDS | 133±15.0 | 195±84.9 | 166±67.5 | 209±69.1 | 241±54.6 |
|  | G-CSF | 140±22.1 | 197±36.3 | 213±24.6 | 273±43.8 | 248±78.7 |
|  | iNO | 135±25.3 | 223±49.7 | 216±39.9 | 267±75.1 | 268±24.5 |

Values are means ± SD.
