# Supplementary material for: Inhaled NO Contributes to Lung Repair in Piglets with Acute Respiratory Distress Syndrome via Increasing Circulating Endothelial Progenitor Cells
Source: PLoS One. 2012 Mar 20;7(3):e33859. doi: 10.1371/journal.pone.0033859 (PMC3309020; doi:10.1371/journal.pone.0033859)
Supplement: Table S2 — The number of EPCs/MNC in peripheral blood at different time points. (DOC) [file pone.0033859.s002.doc]

Table S2: The number of EPCs/MNC in peripheral blood at different time points

|  | Group | B (n=6) | 0 h (n=6) | 24 h (n=6) | 72 h (n=5-6) | 168 h (n=5-6) |
| --- | --- | --- | --- | --- | --- | --- |
| CD34^+^KDR^+^ cells/MNC, % | Con | 0.042±0.013 | 0.034±0.006 | 0.041±0.012 | 0.029±0.014 | 0.026±0.012 |
|  | ARDS | 0.042±0.012 | 0.049±0.027 | 0.041±0.018 | 0.024±0.013 | 0.031±0.015 |
|  | G-CSF | 0.037±0.008 | 0.039±0.007 | 0.036±0.011 | 0.063±0.018* | 0.071±0.018* |
|  | iNO | 0.041±0.013 | 0.036±0.006 | 0.054±0.012* | 0.047±0.012* | 0.043±0.017 |
| CD34^+^KDR^+^CD133^+^ cells/MNC, % | Con | 0.017±0.012 | 0.018±0.011 | 0.020±0.010 | 0.016±0.013 | 0.014±0.007 |
|  | ARDS | 0.018±0.009 | 0.023±0.014 | 0.021±0.013 | 0.013±0.014 | 0.013±0.015 |
|  | G-CSF | 0.016±0.006 | 0.016±0.007 | 0.013±0.006 | 0.018±0.003 | 0.019±0.003 |
|  | iNO | 0.019±0.010 | 0.017±0.006 | 0.028±0.011 | 0.016±0.005 | 0.014±0.011 |
| KDR^+^CD133^+^ cells/MNC, % | Con | 0.020±0.014 | 0.021±0.013 | 0.020±0.012 | 0.016±0.007 | 0.015±0.011 |
|  | ARDS | 0.021±0.007 | 0.025±0.016 | 0.023±0.014 | 0.017±0.014 | 0.016±0.013 |
|  | G-CSF | 0.017±0.007 | 0.018±0.006 | 0.015±0.006 | 0.019±0.003 | 0.027±0.004* |
|  | iNO | 0.020±0.011 | 0.019±0.011 | 0.032±0.011 | 0.019±0.005 | 0.022±0.006 |

Values are means ± SD. MNC, mononuclear cells. * *P* < 0.05 vs. ARDS.
